# Evaluation of Antioxidant and Anti-Inflammatory Activity of Anthocyanin-Rich Water-Soluble Aronia Dry Extracts

**DOI:** 10.3390/molecules25184055

**Published:** 2020-09-04

**Authors:** Mariusz Banach, Magdalena Wiloch, Katarzyna Zawada, Wojciech Cyplik, Wojciech Kujawski

**Affiliations:** 1 Greenvit Ltd., 27A Wojska Polskiego Avenue, 18-300 Zambrów, Poland; mariusz.banach@greenvit.pl (M.B.); wojciech.cyplik@greenvit.pl (W.C.); 2 Faculty of Chemistry, Nicolaus Copernicus University in Toruń, 87-100 Toruń, Poland; 3 AronPharma Ltd., 80-172 Gdańsk, Poland; mlwiloch20@gmail.com; 4 Faculty of Pharmacy with the Laboratory Medicine Division, Medical University of Warsaw, 02-097 Warsaw, Poland; katarzyna.zawada@wum.edu.pl; 5 Faculty of Chemistry and Chemical Technology, Al-Farabi Kazakh National University, 050040 Almaty, Kazakhstan

**Keywords:** *Aronia melanocarpa*, chokeberry, polyphenols, anthocyanins, antioxidant, anti-inflammatory

## Abstract

Aronia fruits contain many valuable components that are beneficial to human health. However, fruits are characterized by significant variations in chemical composition dependent on the growing conditions and harvesting period. Therefore, there is a need to formulate the extracts with a precisely defined content of health-promoting substances. Aronia dry extracts (ADE) were prepared from frozen pomace applying water extraction, followed by purification and spray-drying. Subsequently, the content of anthocyanins, phenolic acids, and polyphenols was determined. The high-quality chokeberry pomace enabled obtaining extracts with anthocyanin content much higher than the typical market standards. Moreover, it was found that the antioxidant capacity of aronia extracts exceeded those found in other fruit preparations. Antioxidant and free-radical scavenging properties were evaluated using a 2,2′-diphenyl-1-picrylhydrazyl using Electron Paramagnetic Resonance (EPR) spectroscopy (DPPH-EPR) test and Oxygen Radical Absorbance Capacity (ORAC) assay. The inhibition of lipid peroxidation and the level of inflammatory markers have been also investigated using lipopolysaccharide (LPS)-stimulated RAW 264 cells. It was revealed that ADE standardized to 25% of anthocyanins depresses the level of markers of inflammation and lipid peroxidation (Interleukin 1 beta (IL-1β), tumor necrosis factor alpha (TNF-α), and malondialdehyde (MDA)) in in vitro conditions. Additionally, it was confirmed that ADE at all analyzed concentrations did not show any cytotoxic effect as demonstrated by 3-(4,5-dimethylthiazol-2-yl)-2,5-diphenyltetrazolium bromide (MTT) assay.

## 1. Introduction

The plant-based nutrients and phytochemicals from various herbs, plants, and fruits are widely used nowadays as health-protecting food additives [1,2,3,4,5]. Aronia (*Aronia melanocarpa* (Michx.) Elliott, chokeberry) is a shrub native to North America (USA and Canada) belonging to the *Rosaceae* family. Aronia was successfully introduced to Europe at the beginning of 20th century. Interestingly, aronia shrubs do not require special chemical protection during the growing period; therefore, the fruits do not contain traces of plant protection chemicals. Aronia berries are used as a food ingredient and also became an important component in the herbal medicine. The health benefits of aronia fruits are intensively studied in numerous laboratories [6,7,8,9].

Aronia fruits are very tart due to the high content of tannins and thus they are not often consumed raw. Therefore, these fruits are the most often processed into juices, jams, and/or concentrates. The processing of aronia berries requires treatment with high temperature to control the microbiological parameters. Concurrently, the thermal processing causes the loss of significant part of bioactive compounds present in berries, in particular anthocyanins [10,11]. The diminution of bioactive compounds level occurs also during storage because of the high water content in the fresh aronia fruits. These undesirable processes can be limited if the dry extracts are prepared from fruits. However, most available aronia extracts are not or only partially soluble in water, which makes them less comfor to be used by consumers. In this work, we have prepared and studied the new water-soluble extract from chokeberry fruit pomace. Currently, manufacturers of plant extracts try to standardize the herbal products to the fixed concentrations of selected compounds [12,13,14,15]. The commonly accepted content of berry fruit extracts in the standardized products is equal to 25% of anthocyanins [12].

Aronia berries have higher polyphenols (in particular anthocyanins) content than most other fruit. Berries contain up to 3 g of polyphenols and 0.3–0.8 g of anthocyanins per 100 g of fresh fruits. High polyphenols content is associated with a very high antioxidant activity of fruits. The following cyanidin glycosides were identified as the anthocyanins of aronia: 3-galactoside, 3-glucoside, 3-arabinoside, and 3-xyloside with a significant predominance of cyanidin-3-galactoside. Aronia berries contain also large amounts of condensed tannins (proanthocyanidins)—polymers and oligomers composed of flavan-3-ol units—mainly epicatechin. Aronia proanthocyanidins are mostly polymers with more than 10 monomers [16]. Aronia contains also quercetin glycosides, primarily quercetin-3-glucoside, and phenolic acids, predominantly chlorogenic acid and neochlorogenic acid. It was proven that aronia fruits and extracts exhibit strong antioxidative potential in vitro [17,18,19,20,21]. Studies conducted by Zheng and Wang [17] showed that extracts from black chokeberries possess significantly stronger antioxidant activity than extracts obtained from blueberries (*Vaccinium corymbosum* L.), cranberries (*Vaccinium macrocarpon* Aiton), and lingonberries (*Vaccinium vitis-idaea* L.). Kähkönen et al. [22] analyzed 92 various phenol-containing plant extracts. The highest antioxidant activity and the highest total phenolic content were found in aronia and crowberry extracts.

The cardioprotective, antidiabetic, anti-inflammatory, immunomodulatory, antibacterial, and anticancer properties of the chokeberry extracts have been also intensively studied [6,7,8,9,14,18,23,24]. Most of the health-promoting activities of aronia berries is owed to their high antioxidant activity. The cardioprotective and anti-inflammatory properties of aronia were also tested in clinical studies of patients suffering from cardiovascular diseases [6]. Research conducted by Naruszewicz et al. [25] reported the beneficial effects of aronia extract on the cardiovascular system of patients after myocardial infarction. That research has also shown the lowering level of markers of oxidative stress and inflammation of patients taking aronia extract in comparison to the placebo group. Aronia extract caused a significant reduction of serum 8-isoprostane, oxidized low-density lipoprotein (Ox-LDL) levels, as well as high-sensitivity C-reactive protein (hs-CRP) and Monocyte Chemoattractant Protein-1 (MCP-1) levels. The vasoactive properties of aronia anthocyanin-rich extracts were detected using the isolated porcine coronary arterial rings. The administration of aronia extract caused dose-dependent vasorelaxation. One of the mechanisms of vasorelaxation is the neutralization of reactive oxygen species (ROS). ROS are highly reactive and can oxidize various biomolecules including lipids, such as polyunsaturated fatty acids (PUFAs). This phenomenon has been named “oxidative stress” [23]. Low doses of *A. melanocarpa* extract prevent the loss of vasorelaxation caused by ROS [26]. According to Devev et al. [27], the most health benefits of chokeberry are related to the antioxidant properties of its ingredients.

RAW 264.7 cells are macrophages from mouse. These cells can be used for the assay of anti-inflammatory activity [28]. Tumor necrosis factor alpha (TNF-α) and interleukin 1β (IL-β) are some of the mediators of inflammation [29]. Acute inflammation is a normal defensive response of organism to infection, injury, or irritation. However, chronic inflammation is associated with a number of chronic diseases including cancer, cardiovascular diseases, diabetes, arthritis, as well as autoimmune and neurological diseases [30].

The purpose of this study was the preparation and complete evaluation of antioxidant and anti-inflammatory properties of the new on the market and commercially available standardized anthocyanin-rich *Aronia melanocarpa* dry water-soluble extract. The material used was the underutilized pomace, which is a waste from juice production, contrary to the usual approach of using the whole fruit. Thus, our approach enables the production of both juice and high-quality dry extract, maximizing the utilization of fruit.

Most of the health-promoting properties of chokeberry are due to its antioxidant properties. Therefore, the antioxidant activity of new extract both in relation to chemicals and in living cells was evaluated. Furthermore, the anti-inflammatory properties of the aronia pomace extract were assessed for the first time, using the inflammation model of RAW 264.7 cells stimulated with LPS. To the best of our knowledge, it is the first assessment of antioxidant and anti-inflammatory properties of anthocyanin-rich extract obtained from a pomace.

Studies of the activity of the extract are usually performed with the extract obtained from the same batch of fruit. However, as the content of bioactive compounds and antioxidant properties of aronia berries can vary greatly depending on the weather conditions and the harvest period [31,32], it was decided to check the variability of the properties of extracts prepared from batches of pomace obtained from fruits harvested during two consecutive years. It is important, especially once the standardization of the commercially produced extract is needed, e.g., for the clinical studies.

## 2. Materials and Methods

### 2.1. Chemicals

Caffeic and chlorogenic acids, lipopolysaccharide (LPS from E. coli 0111:B4), Dulbecco’s Modified Eagle Medium (DMEM), heat-inactivated fetal bovine serum (FBS), and 3-(4,5-dimethylthiazol-2-yl)-2,5-diphenyltetrazolium bromide (MTT) were purchased from Sigma-Aldrich (St. Louis, MO, USA), cyanidin-3-O-glucoside was provided by PhytoLab (Dutendorfer, Germany). A cyanidin monoglycosides mixture containing cyanidin: β-glucopyranoside, β-galactopyranoside, and a-arabinopyranoside was delivered from Polyphenols AS (Sandnes, Norway). Acetonitrile and sodium carbonate were delivered from Honeywell (Morris Township, NJ, USA), formic acid was provided by Merck (Darmstadt, Germany). The Folin–Ciocâlteu reagent and methanol were purchased from Chempur (Piekary Śląskie, Poland). All reagents and solvents were of analytical or HPLC grade and were used as received.

### 2.2. Plant Material

The aronia dry extract was produced according to the technology developed at Greenvit Ltd. This technology is the company’s intellectual property, and some information, e.g., important details about the extraction process or the type of resin used for the separation of extracts cannot be disclosed.

Frozen aronia pomace was thawed, extracted, and filtered. Subsequently, the extracts containing ca. 400 g of anthocyanins were loaded on a chromatography column filled with 20 L of adsorption resin. Then, the column was washed with 100 L of water, and the adsorbed compounds were eluted using 40 L of 75% ethanol. The main fraction from the column was concentrated applying a vacuum evaporator to 30% of dry weight. Afterwards, the extracts were dried on a disk spray dryer (Changzhou Fengqi Drying Equipment Co. Ltd., Zhenglu Town, China) without carrier. Two batches (B1 and B2) of the dried aronia extract were prepared. Batches B1 and B2 were produced from frozen *A. melanocarpa* pomace from fruits harvested in 2016 and 2017, respectively. In order to determine the anthocyanin content in the pomace, 2 g of raw material was homogenized in 50% methanol with 2% acetic acid. The solutions were mixed for 30 min using a magnetic stirrer, filtered, and made up to 100 mL with the same solvent in volumetric flasks. Then, the same procedure was followed for the determination of anthocyanins content in the extracts by applying UV-VIS.

### 2.3. Total Phenolic Content

The total phenolic content was determined utilizing the Folin–Ciocâlteu method with caffeic acid as the standard, according to the procedure described elsewhere [33,34] with some minor modifications. Accurately weighed 0.1 g of extract was dissolved in 50 mL of 80% methanol. Subsequently, 1 mL of extract was added to 4 mL of water and mixed with 0.5 mL of the Folin–Ciocâlteu reagent. After 1 min, 2 mL of 20% (*w*/*v*) sodium carbonate aqueous solution was added and then filled up with water to a mark in a 10 mL volumetric flask. The samples were incubated at room temperature in the dark for 30 min. Then, the absorbance of the solution was measured spectrophotometrically at 760 nm using a UV-1800 spectrophotometer (Shimadzu Corp., Kyoto, Japan). All analyses were carried out in triplicates. The total phenolic content was expressed as a caffeic acid equivalent using a 5-point calibration curve.

### 2.4. Identification and Quantification of Anthocyanins and Phenolic Acids by HPLC-DAD Method

The identification and quantification of anthocyanins and phenolic acids of aronia extract was carried out using a Shimadzu High-Performance LC system equipped with a photodiode array detector (Shimadzu Corp., Kyoto, Japan). The solid extracts were dissolved in 80% methanol acidified with 1% formic acid to obtain the concentration of ca. 1 mg/mL.

The separations were carried out by injecting a 10 µL sample onto a Kinetex Evo C18 column (Phenomenex, Torrance, CA, USA) at 25 °C. The mobile phase consisted of solvent A (4.5% formic acid, *v*/*v*) and solvent B (100% acetonitrile). The isocratic elution started with 5% of solvent B (0–5 min), and then a linear gradient 5–8% B in 5–15 min, 8–20% B in 15–40 min, 20–25% B in 40–50 min, and 25–50% B in 50–55 min was applied. At 55–58 min, the gradient returned to the initial composition (5% B), and subsequently, it was held constant for the additional 8 min to equilibrate the column. The flow rate was equal to 1 mL/min (0–15 min and 50–65 min) and 0.8 mL/min (15–50 min). The calibration curve for cyanidin-3-glucoside chloride (C3G) was generated using five concentration levels. The anthocyanins content was calculated as C3G equivalent. Phenolic acids (chlorogenic and neochlorogenic) contents were expressed as a chlorogenic acid equivalent using a 5-point calibration curve and expressed as chlorogenic acid equivalent. Anthocyanins and phenolic acids were identified on the basis of the characteristic UV-Vis spectrum. Anthocyanins have an absorbance maximum at about 520 and about 280 nm. In turn, chlorogenic and neochlorogenic acids have a maximum at 325 nm. Aronia also has a characteristic anthocyanin and phenolic acids profile facilitating the identification of peaks. An additional confirmation of the identity of the analyzed anthocyanins was a mixture of 3 cyanidin monoglycosides.

### 2.5. Quantification of Anthocyanins by UV-VIS Method

Anthocyanins were also assayed according to the protocol described in detail elsewhere [35]. Prior to the measurements, three samples of dried extract were dissolved in 80% methanol to obtain the concentration of 1 mg/mL. Subsequently, each solution was diluted with buffers pH 1.0 and pH 4.5 in 10-mL volumetric flasks. The flasks were incubated at room temperature in the dark for 20–50 min. The absorbance was measured spectrophotometrically at 520 nm and 700 nm utilizing a 160A UV-1800 spectrophotometer (Shimadzu Corp., Kyoto, Japan). The anthocyanin content was calculated using the molar absorbance coefficient and molecular weight of cyanidin-3-glucoside. Each assay was done in triplicate.

### 2.6. DPPH Scavenging Activity (Electron Paramagnetic Resonance Test)

The free radical scavenging activity of the extracts has been measured in vitro by 2,2′-diphenyl-1-picrylhydrazyl using Electron Paramagnetic Resonance (EPR) spectroscopy (DPPH-EPR) assay following a method described in detail elsewhere [36]. The EPR spectroscopy has been chosen as the anthocyanins-rich extracts absorb in the same wavelength region as DPPH radicals. Thus, for commonly used spectrophotometric measurements, the background corrections for absorbance would be necessary, while in EPR, only the free radical signal is registered. Briefly, 50 μL of an extract solution (in methanol, 0.2 mg/mL) or pure methanol (as a blank) was mixed with 250 μL of 1.3 mM DPPH solution and kept for 40 min in the dark. Then, the content of remaining a DPPH was determined by applying EPR spectroscopy. The EPR spectra were recorded using Miniscope MS200 spectrometer (Magnettech GmbH, Berlin, Germany) with the following parameters: central field 334 mT, sweep range 8 mT, sweep time 30 s, microwave power 10 mW, modulation amplitude 0.1 mT [36]. Results were expressed as micromoles of Trolox per 1 g of extract, using a previously prepared calibration curve. All experiments were done in triplicate.

### 2.7. Oxygen Radical Absorbance Capacity (ORAC Assay)

The ORAC method is based on the oxidative degradation of the fluorescent molecules after being mixed with a free radical generator, for example azo compounds [37]. This method determines the ability of the sample to neutralize short-living free radicals. The ORAC-FL assay was executed following the method proposed by Ou et al. [37] with some minor modifications. Prior to the measurements, the dried extracts (B1 or B2) were dissolved in acidified methanol to obtain the concentration of 1 mg/mL. Subsequently, the methanolic solutions were diluted with PBS (phosphate-buffered saline, pH = 7.4). The dilution was 1000-fold for the B1 batch and 1500 fold for the B2 one. Then, 30 µL of an extract solution or blank (PBS) and 180 µL of 112 nM fluorescein solution were mixed in a well of a 96-well plate and incubated at 37 °C for 15 min. Next, 100 µL of 100 mM AAPH (2,2′-azobis(2-amidinopropane) dihydrochloride) solution was added, and the fluorescence was measured every 70 s for 90 min using an F-7000 Fluorescence Spectrophotometer (Hitachi Ltd., Tokyo, Japan) equipped with a microplate reader. The excitation wavelength was equal to 485 nm, and the emission wavelength was equal to 520 nm. All stock solutions and dilutions of samples were prepared fresh daily in phosphate-buffered saline (PBS, pH 7.4). The experiments were done in six repetitions. Results were expressed as micromoles of Trolox per 1 g of extract [38].

### 2.8. In Vitro Anti-Inflammatory Activity

One of the typical standards of anthocyanins-rich extracts available at the market is the extract standardized for 25% anthocyanins content [12]. Maltodextrin is a typical carrier used for drying and standardizing extracts. Consequently, for the tests with cell cultures, the standardized extract was prepared from batch B1 by adding 2.424 g of maltodextrin to 7.576 g of extract. In order to add the extract to cell cultures, the extract should be dissolved and sterilized. The extract is soluble in water, but it is difficult to initially wet it with water. Therefore, it was moistened with 50% ethanol during preparation of the extract. Then, 2.5 g of extract was accurately weighed, moistened, and afterwards dissolved in water. The volume was made up with water in a 25-mL volumetric flask. The flask was incubated for 10 min in an ultrasonic bath to thoroughly dissolve the extract. Then, the solution was filtered through a 0.2-µm syringe filter for the sterilization. The dissolved and filtered extract was added at an appropriate quantity to the cell culture.

RAW 264.7 cells Cat. No. 91062702, Sigma-Aldrich (St. Louis, MO, USA) were cultured in DMEM supplemented with 10% heat-inactivated FBS, 100 U/mL penicillin, and 100 μg/mL streptomycin. Cells were grown under constant humidity at 37 °C in an atmosphere containing 95% air and 5% CO_2_ for 96 h.

MTT assay was used to determine cytotoxicity—the relative amount of viable cells that are able to convert yellow soluble MTT to purple formazan crystals. Cells (2 × 104 cells per well) were incubated for 24 h at 37 °C, without any addition of the extract (as a control) and with an addition of various amounts of the extract (in the range 0.5–500 µg/mL). Afterwards, the medium was removed from the wells; then, cells were washed with phosphate-buffered saline. Subsequently, the cells were incubated with serum-free medium and MTT (0.5 mg/mL, 100 µL/well) for 4 h. The viable cell number was proportional to the production of water-insoluble formazan. The absorbance was measured spectrophometrically at 563 nm after the dissolution of formazan crystals in isopropanol [39].

In order to determine the influence of the extract on the inflammatory response of RAW 264.7 cells, the various concentrations of extract—0.5, 5, and 500 μg/mL—were added to the cell culture. The cells were stimulated with 1 µg/mL of LPS (from E. coli 0111:B4) for 24 h to polarize macrophages toward the pro-inflammatory subtype and to induce an inflammatory response [38].

Cytokines (TNF-α and Il-1β) levels were quantified by the ELISA method according to the manufacturer’s instructions (R&D Systems Inc., Abingdon, United Kingdom). The malondialdehyde (MDA) level was assayed using a Lipid Peroxidation (MDA) Assay Kit by the competitive ELISA method according to the manufacturer’s instructions (Abcam, Cambridge, MA, USA).

### 2.9. Statistical Analysis

The results of tumor necrosis factor α (TNF-α), interleukin 1 beta (IL-1β), and MDA assays were expressed as mean ± standard error of the mean (S.E.M.) from the three experiments. Significant differences (*p* < 0.05) were determined using the Tukey’s test at a level of α = 0.05. The homogeneity of variance was checked by Levene’s test. Statistical significance analysis was performed using the Origin 2018b software (OriginLab Corporation; Northampton, MA, USA). The charts were prepared using the Grapher 16 program.

## 3. Results and Discussion

### 3.1. Analysis of Aronia Extracts

Quantitative analysis of extracts by the HPLC method revealed the presence of anthocyanins in the both investigated batches of extracts (B1, B2) equal to 33.1% and 14.47%, respectively (Table 1). Alternatively, it was found that the difference in the content of polyphenols (65.48%—B1, 57.45%—B2) and phenolic acids (5.68%—B1, 6.24%—B2) was relatively small. Regardless of the analytical method used (UV-VIS or HPLC), the results of quantitative analysis of anthocyanins are similar for a given batch. The different content of anthocyanins in the extracts from the B1 and B2 batches results from their different content in the raw material. This indicates the great importance of the quality of the raw material used to the standardized extract production. In the raw material used to produce batch B1 (pomace B1), the anthocyanins content amounted to 1.02%, while it was equal to 0.61% in pomace B2. The fruits from which the juice was pressed came from the plantations located in the northeastern part of Poland. The content of anthocyanins in chokeberry fruits has often been investigated, but there is little information on the impact of weather conditions on the content of these substances. It was found that the content of phenolic compounds including anthocyanins in chokeberry fruits is higher in the years with hot and dry weather [31,32]. Summer in 2016 in northeastern Poland was warmer than in 2017. A similar relationship was found for blackcurrant fruit [40]. In the case of grape fruit, it has been found that the anthocyanin content increases with a decrease in available soil water [41]. It is also worth mentioning that the level of anthocyanins strongly depends on the harvesting period [42]. In batch B1, it was much higher than the 22.3% reported by Walther and Müller for the extracts obtained from an aronia pomace; although for B2, it was lower than reported by the same group [43]. In addition, the extract obtained by these researchers due to extraction with a strong acid solution was characterized by a high content of anthocyanin aglycone: cyanidin. Almost one-third of anthocyanins are in the form of an aglycone. Still, the anthocyanin content for the B1 extract has the highest concentration reported to date.

Interestingly, dry extract from the B2 batch, which possesses a lower content of anthocyanins and polyphenols, is characterized by a much higher (1.4–1.7 times) antioxidant activity (Table 1). Explanation of these results will require further research, e.g., by detailed analyses of the remaining extract components.

The results of antioxidative tests suggest the presence of strongly scavenging compounds, which react with both the stable DPPH radical and the peroxyl radical produced in the reaction environment of the ORAC method (Table 1).

**Figure 1:**
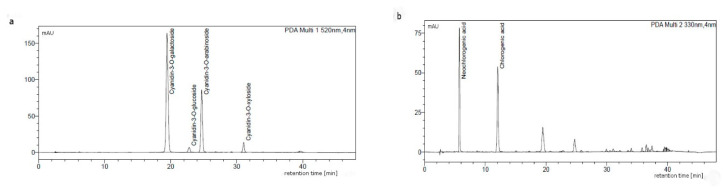
Composition of chokeberry dry extract from batch B1, separated by using HPLC recorded at (**a**) 520 and (**b**) 330 nm.

The anthocyanin and the phenolic acids profiles are consistent with the literature data [16,19,44]. The chromatographic profile of anthocyanins was in both cases characteristic for the chokeberry anthocyanins and showed the presence of four cyanidin glycosides, i.e., 3-galactoside, 3-glucoside, 3-arabinoside, and 3-xyloside (Figure 1a). Extracts contained also chlorogenic and neochlorogenic acids (Figure 1b), which are the main phenolic acids of various cultivars and wild *A. melanocarpa*.

Kähkönen et al. [22] prepared chokeberry fruit extract using 70% acetone as a solvent and the SPE (Solid Phase Extraction) as the purification step. The authors obtained a dry extract with very low polyphenol content, ca. 4% only, as determined by the Folin–Ciocâlteu method. However, the authors did not analyze the content of anthocyanins in the prepared extract. Wang and coworkers [45] utilized extraction with acidified 70% ethanol and two-stage anthocyanin purification using two resins (Amberlite XAD-7 and Sephadex LH-20) obtaining a chokeberry extract containing ca. 50% anthocyanins. The ORAC assay of the extract prepared by these authors gave the results of about 4000 µmol TE/g, whereas the DPPH result exceeded 5000 µmol TE/g. Despite the use of acetone extraction, two-stage separation, and a higher content of anthocyanins in the extract, the obtained values were lower than in our research (Table 1).

Polyphenols, including anthocyanins, are usually extracted with mixtures of water and organic solvents such as ethanol, methanol, or acetone. By using water as a solvent, the new extract is more easily soluble in water, which may increase the bioavailability of polyphenols. Solubility in water is one of the main factors affecting the bioavailability of polyphenols [46]. For example, this also extends the possibilities of its use in functional beverages. According to Polish patent, the aqueous solutions of sulfates (IV) are other efficient anthocyanin extractants [47]. The extracts obtained as a result of this extraction method are also highly water-soluble. The main drawback of this extraction method is sulfite residue present in the extract even after chromatographic purification. Sulfite residue in the extract is unfavorable because of their allergenicity [48].

### 3.2. Cytotoxicity Assay

The MTT tetrazolium dye test is widely used to test the cytotoxicity of the tested substances before any experiments involving cell lines [7,49,50]. The MTT test results are shown in Table 2. The results obtained for various concentrations of extracts are similar to the control sample (0.277), which revealed that the tested product (aronia dry extract) is safe for RAW264 cells at all analyzed concentration levels. No statistically significant differences were found for any of the analyzed extract concentrations compared to the control sample (Table 2).

### 3.3. Effect of Aronia Extract Concentrations on Tumor Necrosis Factor α (TNF-α) and Interleukin 1β (IL-1β) in LPS-Stimulated RAW 264.7 Cells

**Figure 2:**
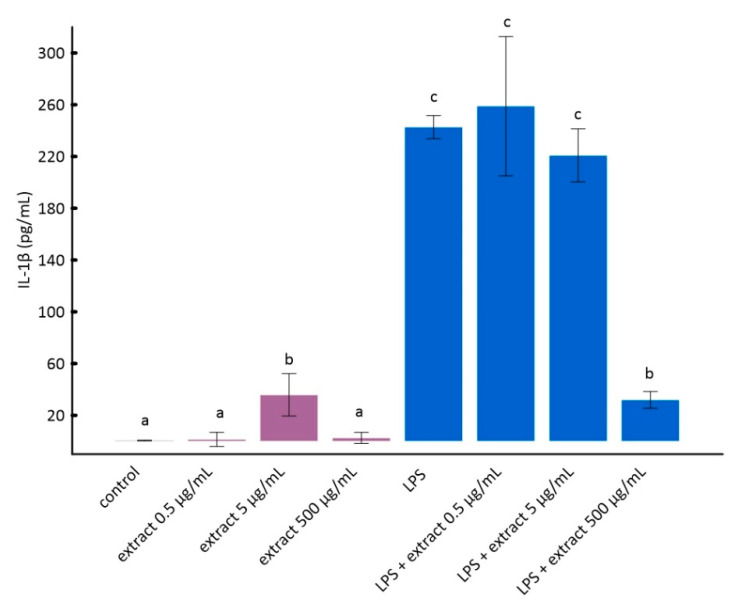
The influence of aronia extract on lipopolysaccharide-induced production of interleukin 1β. The values are means ± SD. The same letter (a, b, and c) indicates that there is no statistically significant difference between the analyzed samples at a confidence level of *p* < 0.05. The different letter indicates the significant difference (*p* < 0.05) among the mean values. The results are ordered as follows: a < b < c.

**Figure 3:**
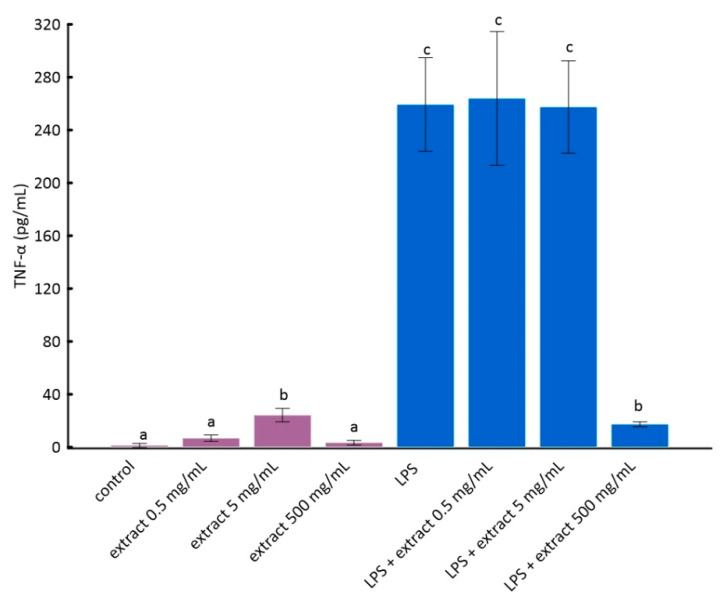
The influence of aronia extract on lipopolysaccharide-induced production of tumor necrosis factor α. The values are means ± SD. The same letter (a, b, and c) indicates that there is no statistically significant difference between the analyzed samples at a confidence level of *p* < 0.05. The different letter indicates the significant difference (*p* < 0.05) among the mean values. The results are ordered as follows: a < b < c.

According to the literature data, pure anthocyanins possess anti-inflammatory properties in vitro and in vivo [51,52]. The results of the analyses are presented in Figure 2 and Figure 3.

The stimulation of RAW 264.7 cells by LPS significantly increases the production of inflammatory factors interleukin 1 beta (IL-1β) and tumor necrosis factor alpha (TNF-α) [28]. As it can be seen from the data in Figure 2 and Figure 3, this increase was much smaller for cells to which ADE was added at concentration of 500 µg/mL. However, among tested concentrations of black chokeberry extract, only 500 µg/mL caused a significant decrease of both inflammation markers compared to LPS-treated cells. The differences between the control and cells treated with aronia extract at a concentration of 500 µg/mL of extract were in both cases statistically non-significant. The concentrations of 0.5 µg/mL and 5 µg/mL did not change the level of any of the analyzed markers. Anthocyanin-rich extract alone caused a slight but not statistically significant increase in the secretion of TNF-α and IL-1β (Figure 2 and Figure 3).

Similar effects were observed in the studies of Lee and co-workers [7] on the immortalized murine microglial cell line BV2 cells. In their studies, it was shown that aronia extract reduced the mRNA expression levels of LPS-induced inflammatory signals in BV2 cells at a concentration of 300 μg/mL [7]. The anti-inflammatory effect of chokeberry extract has been confirmed also in the human aortic endothelial cells. *A. melanocarpa* extract inhibits the expression of cell adhesion molecules and nuclear factor NF-κB (nuclear factor kappa-light-chain-enhancer of activated B cells) in these cells. Aronia extract inhibited also tumor necrosis factor TNF-α-induced intracellular reactive oxygen species generation [44]. In a study conducted by Martin and coworkers [49], it was shown that extracts from various species of chokeberry, despite significant differences in their composition, similarly inhibit LPS-induced interleukin-6 (IL-6) secretion by murine splenocytes.

Interesting results were obtained by Xu and Mojsoska, who prepared raw chokeberry extract and anthocyanin-rich extract [53]. In their study, the immunomodulatory activity of raw chokeberry extract and the purified anthocyanin-rich fraction (AF) was compared. The crude extract inhibited the production of IL-6 by mocytes, which is a pro-inflammatory cytokine that inhibits the production of pro-inflammatory TNF-α. On the other hand, AF showed no statistically significant immunomodulatory activity.

Lee and others [54] compared the anti-inflammatory and antioxidant properties of red clover flower extract as well as anthocyanins isolated from the same raw material. They have proven that both the crude extract and anthocyanins possess antioxidant and anti-inflammatory effects. Interestingly, red clover anthocyanins did not cause a decrease in TNF-α, but they did cause a decrease in IL1-ß.

### 3.4. Lipid Peroxidation MDA Assay

Malondialdehyde (MDA) is one of the commonly assayed markers of oxidative stress. This is a product of polyunsaturated fatty acids (PUFAs) peroxidation [55]. This analysis was carried out to check whether the antioxidant activity of the chokeberry extracts demonstrated by ORAC and DPPH tests is confirmed in cell cultures.

**Figure 4:**
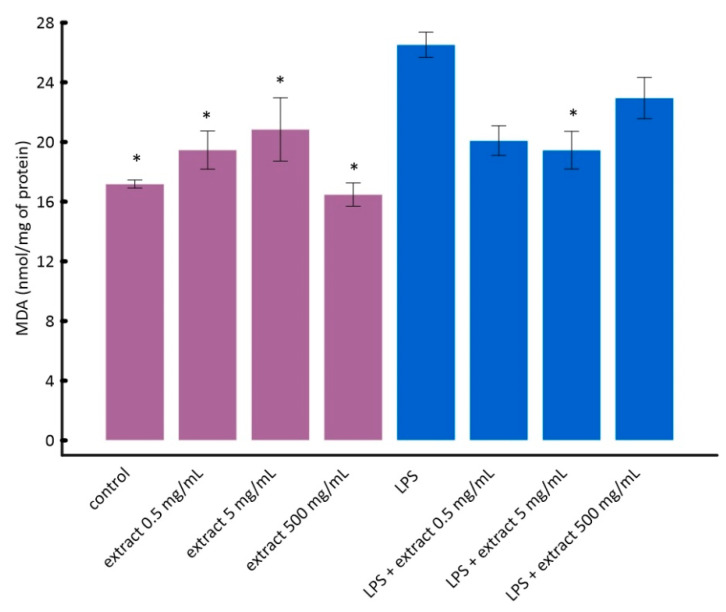
The influence of aronia extract on lipopolysaccharide-induced lipid peroxidation. The values are means ± SD. Results for samples marked with * do not differ statistically from control cells at *p* < 0.05.

It can be seen in Figure 4 that LPS caused a statistically significant increase in lipid peroxidation in vitro. Black chokeberry extract attenuated this effect. Interestingly, lower concentrations of chokeberry extract inhibited lipid peroxidation stronger than higher concentrations (Figure 4). However, the differences between cells treated with LPS and aronia extract at different concentrations were not statistically significant. This is consistent with the results of other researchers showing that anthocyanin-rich extracts, e.g., açaí or purple sweet potato, may have a pro-oxidative effect at high concentrations [56,57].

In the research carried out by Kardum et al. [58] on the healthy volunteers, it was found that polyphenol-rich aronia juice significantly decreased lipid peroxidation assayed as thiobarbituric acid-reactive substances (TBARS). Other extracts and juices that were rich in anthocyanins (such as bilberry, black currant, and elderberry) also caused a significant reduction in the oxidative stress level in vitro and in vivo [59,60,61].

## 4. Conclusions

It is known that the *Aronia melanocarpa* fruits prevent some chronic diseases, primarily cardiovascular ones, which is associated with the antioxidative and anti-inflammatory properties of these fruits. Up to now, the scientific publications most commonly presented investigations on the properties of the extracts obtained uniquely from chokeberry and other berry fruits. This work shows that valuable, water-soluble extracts can be produced from the pomace, which is regarded as a perishable waste material. Two pilot batches of the novel, water-soluble chokeberry anthocyanin-rich extracts were produced for the first time using the aronia pomace as a source material. The two batches of extract, despite the same production method, differed significantly regarding the composition, mainly in anthocyanins content, antioxidant properties measured with DPPH-EPR and ORAC tests, and anti-inflammatory properties. The only difference in the production of both batches of extract was the source of the raw material. This difference in composition of the extracts shows that selection of the high-quality raw material in the production of a standardized extract is important. Nevertheless, the content of polyphenols and phenolic acids in both batches of extract were at a similar level.

The antioxidant and anti-inflammatory activity of ADE from batch B1 of pomace was demonstrated. Using RAW 264.7 cells, a reduction in inflammation markers has been shown as well as a lack of cytotoxicity of the extract. The extract shows antioxidant activity against chemicals (DPPH and ORAC methods) and in the contact with cells by causing the decrease of lipid peroxidation in vitro. Thus, the dry extract from the aronia pomace is a promising substrate for functional food products and clinical studies.

## Figures and Tables

**Table 1:** Antioxidant activity and the content of marked components of *Aronia melanocarpa* extracts. DPPH: 2,2-diphenyl-1-picrylhydrazyl, ORAC: Oxygen Radical Absorbance Capacity.

Batch ID	Total Polyphenols Calculated as Caffeic Acid (UV_VIS) [*wt*%]	Anthocyanins Calculated as Cy-3-glu Chloride (HPLC) [*wt*%]	Anthocyanins Calculated as Cy-3-glu (UV-VIS) [*wt*%]	Phenolic Acids Calculated as Chlorogenic Acid (HPLC) [*wt*%]	DPPH (Trolox Equivalent) [µmol TE/g]	ORAC (Trolox Equivalent) [µmol TE/g]
B1	65.48 ± 1.86	33.10 ± 0.28	32.54 ± 0.28	5.682 ± 0.114	6417 ± 93	10,519 ± 150
B2	57.48 ± 1.55	14.47 ± 0.03	16.58 ± 0.13	6.247 ± 0.171	11,127 ± 219	15,335 ± 452

**Table 2:** 3-(4,5-dimethylthiazol-2-yl)-2,5-diphenyltetrazolium bromide (MTT) cell viability assay results.

	Sample			
	Control	Extract 0.5 µg/mL	Extract 5 µg/mL	Extract 500 µg/mL
MTT test results (as A_563_)	0.277 ± 0.018	0.279 ± 0.017	0.279 ± 0.009	0.281 ± 0.006

## Symbols and Abbreviations

ADE	Aronia dry extract
C3G	Cyanidin-3-glucoside chloride
DMEM	Dulbecco’s Modified Eagle Medium
DPPH	2,2-Diphenyl-1-picrylhydrazyl
EPR	Electron Paramagnetic Resonance
FBS	Fetal bovine serum
hs-CRP	High-sensitivity C-reactive protein
IL-1β	Interleukin-1 beta
LPS	Lipopolysaccharide
MCP-1	Monocyte Chemoattractant Protein-1
MDA	Malondialdehyde
MTT	3-(4,5-dimethylthiazol-2-yl)-2,5-diphenyltetrazolium bromide
NF-κB	nuclear factor kappa-light-chain-enhancer of activated B cells
ORAC	Oxygen Radical Absorbance Capacity
Ox-LDL	Oxidized low-density lipoprotein
PBS	Phosphate-buffered saline
PUFAs	Polyunsaturated fatty acids
ROS	Reactive oxygen species
SPE	Solid Phase Extraction
SD	Standard Deviation
TNF-α	Tumor necrosis factor α

## References

[1] Ammendola M., Haponska M., Balik K., Modrakowska P., Matulewicz K., Kazmierski L., Lis A., Kozlowska J., Garcia-Valls R., Giamberini M. (2020). Stability and anti-proliferative properties of biologically active compounds extracted from *Cistus* L. after sterilization treatments. Sci. Rep..

[2] Calvani M., Pasha A., Favre C. (2020). Nutraceutical Boom in Cancer: Inside the Labyrinth of Reactive Oxygen Species. Int. J. Mol. Sci..

[3] Montané X., Kowalczyk O., Reig-Vano B., Bajek A., Roszkowski K., Tomczyk R., Pawliszak W., Giamberini M., Mocek-Płóciniak A., Tylkowski B. (2020). Current Perspectives of the Applications of Polyphenols and Flavonoids in Cancer Therapy. Molecules.

[4] Sammar M., Abu-Farich B., Rayan I., Falah M., Rayan A. (2019). Correlation between cytotoxicity in cancer cells and free radical-scavenging activity: In vitro evaluation of 57 medicinal and edible plant extracts. Oncol. Lett..

[5] Sawicka B., Ziarati P., Krochmal-Marczak B., Skiba D. (2019). Nutraceuticals in food and pharmacy. A Review. Agron. Sci..

[6] Kokotkiewicz A., Jaremicz Z., Luczkiewicz M. (2010). Aronia Plants: A Review of Traditional Use, Biological Activities, and Perspectives for Modern Medicine. J. Med. Food.

[7] Lee K.P., Choi N.H., Kim H.S., Ahn S., Park I.S., Lee D.W. (2018). Anti-neuroinflammatory effects of ethanolic extract of black chokeberry (*Aronia melanocapa* L.) in lipopolysaccharide-stimulated BV2 cells and ICR mice. Nutr. Res. Pract..

[8] Sidor A., Drożdżyńska A., Gramza-Michałowska A. (2019). Black chokeberry (*Aronia melanocarpa*) and its products as potential health-promoting factors-An overview. Trends Food Sci. Technol..

[9] Park S., Kim J.I., Lee I., Lee S., Hwang M.W., Bae J.Y., Heo J., Kim D., Han S.Z., Park M.S. (2013). *Aronia melanocarpa* and its components demonstrate antiviral activity against influenza viruses. Biochem. Biophys. Res. Commun..

[10] Walkowiak-Tomczak D. (2007). Changes in antioxidant activity of black chokeberry juice concentrate solutions during storage. Acta Sci. Pol. Technol. Aliment..

[11] Patras A., Brunton N.P., O’Donnell C., Tiwari B.K. (2010). Effect of thermal processing on anthocyanin stability in foods; mechanisms and kinetics of degradation. Trends Food Sci. Technol..

[12] Gardana C., Scialpi A., Fachechi C., Simonetti P. (2018). Near-infrared spectroscopy and chemometrics for the routine detection of bilberry extract adulteration and quantitative determination of the anthocyanins. J. Spectrosc..

[13] Davinelli S., Bertoglio J.C., Zarrelli A., Pina R., Scapagnini G. (2015). A Randomized Clinical Trial Evaluating the Efficacy of an Anthocyanin–Maqui Berry Extract (Delphinol^®^) on Oxidative Stress Biomarkers. J. Am. Coll. Nutr..

[14] Borowska S., Tomczyk M., Strawa J.W., Brzóska M.M. (2020). Estimation of the Chelating Ability of an Extract from *Aronia melanocarpa* L. Berries and Its Main Polyphenolic Ingredients Towards Ions of Zinc and Copper. Molecules.

[15] Scaglione F., Pannacci M., Petrini O. (2005). The standardised G115^®^ Panax ginseng C.A. Meyer extract: A review of its properties and usage. J. Evid. Based Integr. Med..

[16] Wu X., Gu L., Prior R.L., McKay S. (2007). Characterization of Anthocyanins and Proanthocyanidins in Some Cultivars of Ribes, Aronia, and Sambucus and Their Antioxidant Capacity. J. Agric. Food Chem..

[17] Zheng W., Wang S.Y. (2003). Oxygen radical absorbing capacity of phenolics in blueberries, cranberries, chokeberries, and lingonberries. J. Agric. Food Chem..

[18] Dąbrowski A., Onopiuk B.M., Car H., Onopiuk P., Dąbrowska Z.N., Rogalska J., Brzóska M.M., Dąbrowska E. (2020). Beneficial Impact of an Extract from the Berries of *Aronia melanocarpa* L. on the Oxidative-Reductive Status of the Submandibular Gland of Rats Exposed to Cadmium. Antioxidants.

[19] Wangensteen H., Bräunlich M., Nikolic V., Malterud K.E., Slimestad R., Barsett H. (2014). Anthocyanins, proanthocyanidins, and total phenolics in four cultivars of aronia: Antioxidant and enzyme inhibitory effects. J. Funct. Foods.

[20] Janiszewska-Turak E., Sak A., Witrowa-Rajchert D. (2019). Influence of the carrier material on the stability of chokeberry juice microcapsules. Int. Agrophys..

[21] Bednarska M.A., Janiszewska-Turak E. (2020). The influence of spray drying parameters and carrier material on the physico-chemical properties and quality of chokeberry juice powder. J. Food Sci. Technol..

[22] Kähkönen M.P., Hopia A.I., Vuorela H.J., Rauha J.P., Pihlaja K., Kujala T.S., Heinonen M. (1999). Antioxidant activity of plant extracts containing phenolic compounds. J. Agric. Food Chem..

[23] Jurikova T., Mlcek J., Skrovankova S., Sumczynski D., Sochor J., Hlavacova I., Snopek L., Orsavova J. (2017). Fruits of black chokeberry *Aronia melanocarpa* in the prevention of chronic diseases. Molecules.

[24] Lorenzon dos Santos J., Schaan de Quadros A., Weschenfelder C., Bueno Garofallo S., Marcadenti A. (2020). Oxidative Stress Biomarkers, Nut-Related Antioxidants, and Cardiovascular Disease. Nutrients.

[25] Naruszewicz M., Łaniewska I., Millo B., Dłuzniewski M. (2007). Combination therapy of statin with flavonoids rich extract from chokeberry fruits enhanced reduction in cardiovascular risk markers in patients after myocardial infraction (MI). Atherosclerosis.

[26] Bell D.R., Gochenaur K. (2006). Direct vasoactive and vasoprotective properties of anthocyanin-rich extracts. J. Appl. Physiol..

[27] Denev P.N., Kratchanov C.G., Ciz M., Lojek A., Kratchanova M.G. (2012). Bioavailability and Antioxidant Activity of Black Chokeberry (*Aronia melanocarpa*) Polyphenols: In vitro and in vivo Evidences and Possible Mechanisms of Action: A Review. Compr. Rev. Food Sci. Food Saf..

[28] Hwang S.J., Kim Y.W., Park Y., Lee H.J., Kim K.W. (2014). Anti-inflammatory effects of chlorogenic acid in lipopolysaccharide-stimulated RAW 264.7 cells. Inflamm. Res..

[29] Ji H., Pettit A., Ohmura K., Ortiz-Lopez A., Duchatelle V., Degott C. (2002). Critical Roles for Interleukin 1 and Tumor Necrosis Factor α in Antibody-induced Arthritis. J. Exp. Med..

[30] Feghali C.A., Wright T.M. (1997). Cytokines in Acute and chronic inflammation. Front. Biosci..

[31] Tolić M.T., Krbavčić I.P., Vujević P., Milinović B., Jurčević I.L., Vahčić N. (2017). Effects of Weather Conditions on Phenolic Content and Antioxidant Capacity in Juice of Chokeberries (*Aronia melanocarpa* L.). Pol. J. Food Nutr. Sci..

[32] Gralec M., Wawer I., Zawada K. (2019). *Aronia melanocarpa* berries: Phenolics composition and antioxidant properties changes during fruit development and ripening. Emir. J. Food Agric..

[33] Kujawski W., Sobolewska A., Jarzynka K., Güell C., Ferrando M., Warczok J. (2013). Application of osmotic membrane distillation process in red grape juice concentration. J. Food Eng..

[34] Zainol M.K., Abd-Hamid A., Yusof S., Muse R. (2003). Antioxidative activity and total phenolic compounds of leaf, root and petiole of four accessions of *Centella asiatica* (L.) Urban. Food Chem..

[35] Giusti M.M., Wrolstad R.E. (2001). Characterization and Measurement of Anthocyanins by UV-visible Spectroscopy. Curr. Protoc. Food Anal. Chem..

[36] Sanna D., Delogu G., Mulas M., Schirra M., Fadda A. (2012). Determination of Free Radical Scavenging Activity of Plant Extracts Through DPPH Assay: An EPR and UV—Vis Study. Food Anal. Methods.

[37] Ou B., Hampsch-Woodill M., Prior R.L. (2001). Development and Validation of an Improved Oxygen Radical Absorbance Capacity Assay Using Fluorescein as the Fluorescent Probe. J. Agric. Food Chem..

[38] Hambleton J., Weinstein S.L., Lem L., Defranco A.L. (1996). Activation of c-Jun N-terminal kinase in bacterial lipopolysaccharide-stimulated macrophages. Proc. Natl. Acad. Sci. USA.

[39] Berridge M.V., Herst P.M., Tan A.S. (2005). Tetrazolium dyes as tools in cell biology: New insights into their cellular reduction. Biotechnol. Annu. Rev..

[40] Zheng J., Yang B., Ruusunen V., Laaksonen O., Tahvonen R., Hellsten J., Kallio H. (2012). Compositional Differences of Phenolic Compounds between Black Currant (*Ribes nigrum* L.) Cultivars and Their Response to Latitude and Weather Conditions. J. Agric. Food Chem..

[41] Concepción Ramosa M., Pérez-Álvarezb E.P., Peregrinac F., Martínez de Todad F. (2020). Relationships between grape composition of Tempranillo variety and available soil water and water stress under different weather conditions. Sci. Hortic..

[42] Zielińska A., Siudem P., Paradowska K., Gralec M., Kaźmierski S., Wawer I. (2020). *Aronia melanocarpa* Fruits as a Rich Dietary Source of Chlorogenic Acids and Anthocyanins: 1H-NMR, HPLC-DAD, and Chemometric Studies. Molecules.

[43] Wathon M.H., Beaumont N., Benohoud M., Blackburn R.S., Rayner C.M. (2019). Extraction of anthocyanins from *Aronia melanocarpa* skin waste as a sustainable source of natural colorants. Color. Technol..

[44] Jakobek L., Drenjančević M., Jukić V., Šeruga M. (2012). Phenolic acids, flavonols, anthocyanins and antiradical activity of “Nero”, “Viking”, “Galicianka” and wild chokeberries. Sci. Hortic..

[45] Wang Y., Zhao L., Wang D., Huo Y., Ji B. (2016). Anthocyanin-rich extracts from blackberry, wild blueberry, strawberry, and chokeberry: Antioxidant activity and inhibitory effect on oleic acid-induced hepatic steatosis in vitro. J. Sci. Food Agric..

[46] Biasutto L., Marotta E., Bradaschia A., Fallica M., Mattarei A., Garbisa S., Zoratti M., Paradisi C. (2009). Soluble polyphenols: Synthesis and bioavailability of 3,4′,5-tri(α-d-glucose-3-O-succinyl) resveratrol. Bioorg. Med. Chem. Lett..

[47] Han S., Hauzer A. (2000). Sposób otrzymywania barwników antocyjanowych oraz odzyskiwania barwników antocyjanowych z roślinnych odpadów poprodukcyjnych (Method of obtaining anthocyanin dyes and recovering such dyes from vegetal production wastes). Polish Patent.

[48] García-Gavín J., Parente J., Goossens A. (2012). Allergic contact dermatitis caused by sodium metabisulfite: A challenging allergen. A case series and literature review. Contact Derm..

[49] Martin D.A., Taheri R., Brand M.H., Draghi A., Sylvester F.A., Bolling B.W. (2014). Anti-inflammatory activity of aronia berry extracts in murine splenocytes. J. Funct. Foods.

[50] Zapolska-Downar D., Bryk D., Małecki M., Hajdukiewicz K., Sitkiewicz D. (2012). *Aronia melanocarpa* fruit extract exhibits anti-inflammatory activity in human aortic endothelial cells. Eur. J. Nutr..

[51] Sun Y.A.N., Li L. (2018). Cyanidin-3-glucoside inhibits inflammatory activities in human fibroblast-like synoviocytes and in mice with collagen-induced arthritis. Clin. Exp. Pharmacol. Physiol..

[52] Ferrari D., Cimino F., Fratantonio D., Molonia M.S., Bashllari R., Busà R., Saija A., Speciale A. (2017). Cyanidin-3-O-Glucoside Modulates the in Vitro Inflammatory Crosstalk between Intestinal Epithelial and Endothelial Cells. Mediat. Inflamm..

[53] Xu J., Mojsoska B. (2013). The immunomodulation effect of aronia extract lacks association with its antioxidant anthocyanins. J. Med. Food.

[54] Lee S.G., Brownmiller C.R., Lee S.-O., Kang H.W. (2020). Anti-Inflammatory and Antioxidant Effects of Anthocyanins of Trifolium pratense (Red Clover) in Lipopolysaccharide-Stimulated RAW-267.4 Macrophages. Nutrients.

[55] Tsikas D. (2017). Assessment of lipid peroxidation by measuring malondialdehyde (MDA) and relatives in biological samples: Analytical and biological challenges. Anal. Biochem..

[56] Cai Z., Song L., Qian B., Xu W., Ren J., Jing P., Oey I. (2018). Understanding the effect of anthocyanins extracted from purple sweet potatoes on alcohol-induced liver injury in mice. Food Chem..

[57] Santos V.D.S., Bisen-Hersh E., Yu Y., Ribeiro Cabral I.S., Nardini V., Culbreth M., Teixeira la Rocha J.B., Barbosa F., Aschner M. (2014). Anthocyanin-rich açaí (*Euterpe oleracea* Mart.) extract attenuates manganese-induced oxidative stress in rat primary astrocyte cultures. J. Toxicol. Environ. Health Part A Curr. Issues.

[58] Kardum N., Konić-Ristić A., Šavikin K., Spasić S., Stefanović A., Ivanišević J., Miljković M. (2014). Effects of Polyphenol-Rich Chokeberry Juice on Antioxidant/Pro-Oxidant Status in Healthy Subjects. J. Med. Food.

[59] Lyall K.A., Hurst S.M., Cooney J., Jensen D., Lo K., Hurst R.D., Stevenson L.M. (2009). Short-term blackcurrant extract consumption modulates exercise-induced oxidative stress and lipopolysaccharide-stimulated inflammatory responses. Am. J. Physiol. Integr. Comp. Physiol..

[60] Murkovic M., Abuja P.M., Bergmann A.R., Zirngast A., Adam U., Winklhofer-Roob B.M., Toplak H. (2004). Effects of elderberry juice on fasting and postprandial serum lipids and low-density lipoprotein oxidation in healthy volunteers: A randomized, double-blind, placebo-controlled study. Eur. J. Clin. Nutr..

[61] Valentová K., Ulrichová J., Cvak L., Šimánek V. (2006). Cytoprotective effect of a bilberry extract against oxidative damage of rat hepatocytes. Food Chem..

